# Dissecting cancer in a non-mammalian model: genomic insights from lemon frost geckos

**DOI:** 10.1186/s12915-026-02661-0

**Published:** 2026-07-15

**Authors:** Brandon T. Hastings, Tony Gamble, Robert J. Ossiboff, Virginia Gazziero, Giulio Caravagna, Scott Glaberman, Ylenia Chiari

**Affiliations:** 1 School of Biosciences, University of Nottingham, Nottingham, UK; 2 Department of Mathematics, Informatics, and Geosciences, University of Trieste, Trieste, Italy; 3 Department of Biological Sciences, Marquette University, Milwaukee, WI USA; 4 Department of Comparative, Diagnostic, and Population Medicine, College of Veterinary Medicine, University of Florida, Gainesville, FL USA; 5 Centre for Environmental Research and Justice, School of Biosciences, University of Birmingham, Birmingham, UK; 6 School of Life Sciences, University of Nottingham, University Park, Nottingham, UK

**Keywords:** Animal model, Comparative oncology, Copy number variation, Gene fusion, Pigment cell cancer, Reptiles, TATA-box binding protein (TBP)

## Abstract

**Background:**

Comparative oncology can uncover novel mechanisms of cancer resistance and progression across animals. Non-traditional model organisms with naturally high or low cancer prevalence may provide further insights into metastasis or cancer resistance. In this context, the “lemon frost” morph of the leopard gecko (*Eublepharis macularius*) shows high prevalence of iridophoromas, exceeding 80%. These tumors can recur and metastasize, presenting a unique opportunity to study pigment cell cancer and tumor progression in a non-mammalian vertebrate.

**Results:**

We performed high-coverage whole-genome sequencing on matched tumor and non-tumor samples from three lemon frost individuals. A shared missense mutation in the TATA-box binding protein (TBP) suggests possible disruption to transcriptional regulation in tumor samples. Additionally, a recurrent gene fusion between *IARS1* and *RNF213* was identified across all tumor samples. Copy number neutral loss of heterozygosity events were observed in three mutated genes that have a role in cancer: *MAP3K13*, *TENM4*, and *OR2AT4-like*, while copy number gains were observed in four genomic regions. Pathway analysis revealed dysregulation in actin filament organization, a hallmark of metastatic potential. These findings suggest a multifaceted genomic basis for tumorigenesis in this model, including transcriptional misregulation, chromatin remodeling defects, and cytoskeletal disruption.

**Conclusions:**

This study provides the first characterization of genomic changes associated with iridophoroma in a reptilian species and establishes the lemon frost gecko as a promising model for cancer research. Our findings identify candidate pathways of tumor progression and metastasis in a non-traditional system, highlighting both conserved and novel mechanisms relevant to human disease.

**Supplementary Information:**

The online version contains supplementary material available at 10.1186/s12915-026-02661-0.

## Background

Comparative oncology is an emerging field that is reshaping cancer research [1]. While classical animal models such as rodents, zebrafish, and fruit flies have provided valuable insights into cancer biology [2, 3], examining cancer prevalence and resistance across the tree of life offers a broader evolutionary perspective. This approach can uncover naturally occurring pathways of initiation, progression, and suppression that may be overlooked in traditional models, thereby enhancing our understanding of cancer resilience and ultimately informing therapeutic strategies.

A foundational example of how comparative studies can generate new models comes from melanoma development in naturally occurring *Xiphophorus* fish hybrids. These fish played a critical role in early melanoma research [4] because of the strong similarity to human melanoma [5]. Unlike mouse models, where melanoma is restricted to hair follicles within the dermis, which differs from human disease, melanomas in *Xiphophorus* develop naturally in the epidermis [6].

More recently, other species have been studied for their potential insights into cancer biology [7, 8]. Turtles (Chelonia), for example, have low cancer prevalence [9], likely due to multiple mechanisms, including slower metabolism, resistance to DNA damage, and enhanced responses to cellular stress [10–12]. The African bush elephant (*Loxodonta africana*) possesses enhanced cancer defenses compared to closely related species due to an increase in TP53 copy numbers [13]. Other long-lived species are currently being investigated for their own potentially unique anticancer mechanisms, such as the Greenland shark (*Somniosus microcephalus*) [14] and Balaenid whales [15]. Additionally, a series of studies have shown that naked mole-rats (*Heterocephalus glaber*) are not only resistant to primary tumor formation, but also to processes leading to metastasis. Naked mole-rats rarely develop tumors even under experimental conditions [16], which is thought to result from a defensive tumor microenvironment [17].

Leopard geckos (*Eublepharis macularius*) are emerging as a promising new model for cancer research. Together with being a popular reptile in the pet trade due to their wide-ranging coloration and ease of captive care, they are also an established system for studying coloration, pattern development, sex determination, and tissue regeneration [18–22]. One of the selectively bred morphs of the leopard gecko—“lemon frost” (LF)—is highly susceptible to tumor development. This morph arose from a spontaneous genetic mutation in a single male and was later stabilized through line breeding [23, 24]. LF geckos are characterized by a lighter body color than wild-type individuals, distinctive frosted white eyes, and a silvery iridescent appearance. They also contain an increased number of iridophores compared to wild-type leopard geckos [23, 24].

**Fig. 1:**
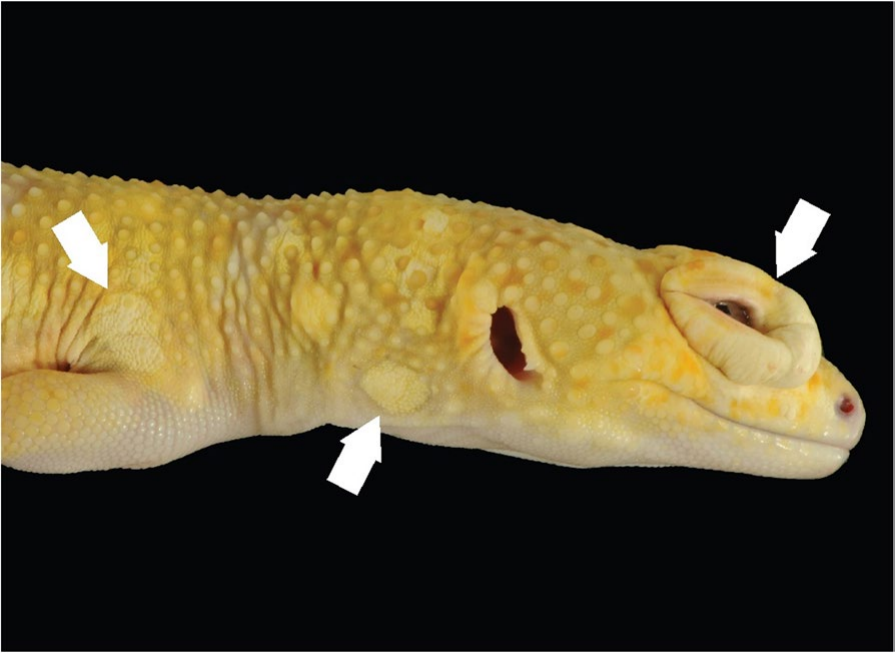
Lemon frost gecko with iridophoroma. Visible iridophoroma masses (indicated by white arrows) on the dermis of individual A23-239

In 80% of cases, LF geckos develop iridophoromas [23], a type of pigment cell neoplasm in reptiles in which iridophores proliferate uncontrolled in the dermis forming dense, white nodules (Fig. 1). Although these tumors may initially be confined to the dermis and removed surgically, they can appear again in new areas over time [24]. Because the independent emergence of multiple primary tumors is rare [25, 26], the widespread skin lesions in LF geckos are likely evidence of metastasis. Metastases are commonly observed in this morph [27], with higher frequency in homozygous individuals at the LF locus [23]. The most frequent site of observed metastases is the liver [23].

Using a large dataset of more than 900 individuals including wild-type leopard geckos, and homozygous and heterozygous at the LF locus and QTL mapping, the LF phenotype has been mapped to SPINT1 [23, 28], a gene also linked to cancer in humans. These studies identified SPINT1 as a strong candidate gene associated with the LF phenotype and the development of iridophoroma in leopard geckos. However, despite the important advances made by Guo et al. [23] indicating SPINT1 as the strongest candidate locus for the LF phenotype, there are no non-synonymous mutations and no differences in mRNA expression when comparing LF and wild-type geckos [23]. Guo et al. [23] proposed that the identified deletions and insertions in SPINT1 promoter and UTR sequences may function similarly as in fish and mice to cause cancer. Therefore, the genetic and genomic variants underlying tumor development and spread in LF geckos remain unresolved. How the tumor evolves and metastasizes remains a critical gap, as metastasis—the spread of cancer cells from a primary tumor to distant organs—drives most cancer-related mortality in organisms [29, 30]. Because the metastatic process involves several steps, including extravasation into new tissues and colonization of secondary sites [31], and every stage represents a barrier to the tumor spread, only a subset of primary tumors ultimately succeeds in forming secondary growths [32]. Given the high frequency of tumors and metastatic lesions observed in LF geckos, elucidating the mechanisms of tumor development in this morph could provide valuable insights into the genetic basis and evolutionary origins of metastasis across organisms.

A major challenge in studying cancer development and spread is the general reliance on classical model species. For example, the most widely used model, *Mus musculus*, does not reflect the human lifespan and often requires transgenic modification or implantation to induce tumors [33]. Other mammals, such as canines, more closely resemble human cancer because they develop spontaneous tumors [34], but their use in research is constrained by dependence on clinical cases, enrichment of high-risk populations (e.g., unspayed females with increased mammary tumors), and uncontrolled environmental variables depending on the source of individuals (e.g., clinical cases, animal shelters). By contrast, new model species from diverse taxa can capture additional features of cancer development and spread at the molecular level. For instance, naturally occurring cancers in amphibians have been shown to mirror human cancer progression, including temperature-dependent metastasis [35], analogous to that seen in human breast cancers [36]. While traditional murine models require transgenic induction to simulate late-stage disease, certain reptilian lineages—specifically the LF morph of leopard geckos—exhibit naturally occurring, aggressive malignancies [23, 24]. The primary advantage of this model lies in its capacity for spontaneous metastasis [23], providing a rare window into the natural progression of the disease that is often difficult to replicate in induced mammalian models.

In this study, we conducted high-coverage whole-genome sequencing (WGS, 100 ×) on primary tumors and matched normal (non-tumor) tissue from three LF individuals to identify the genome-wide mutational impact and broad pathway disruptions of this cancer. Identifying potentially shared and distinct features of tumor development and spread in LF geckos versus other study systems is a critical step toward establishing the LF phenotype as a new model for cancer research that is complementary to traditional animal systems. By generating deep sequencing data from reptilian tumors—a group underrepresented compared to traditional model organisms such as murine rodents, canines, and zebrafish—together with the work by Guo et al. [23], this study establishes a genetic baseline for further investigations into cancer biology in general and iridophoromas.

## Results

### Quality of DNA sequencing

Samples contained a mean depth of coverage between 94.2 × and 159.4 × with only Tumor 3 (Table 1) below 100 × (but above 90 ×); all samples had 99% of the genome with over 30 × coverage. Alignments had between 1597.6 M and 2735.1 M total reads mapped to the leopard gecko genome which represented > 99.7% of reads mapped for each sample, with between 11.5 and 15.5% of those reads being duplicate sequences before filtering via *GATK markduplicates*. GC content was between 43.6 and 44.1% for each sample, independently if they were tumor or non-tumor samples. The genome heterozygosity of normal samples from the current study was 0.34% for A24-050, 0.40% for A23-239, and 0.42% for A23-628 (the individual homozygous for the LF allele), while the heterozygosity of the resequenced pool-seq data from Guo et al. [23] was 0.49%, 0.45%, and 0.46% for wild-type, heterozygous (LF), and homozygous (SLF) populations, respectively.

**Table 1:** Summary of structural variant events for the three tumor samples. “CTX,” “DEL,” “DUP,” “INS,” “INV,” and “TBL” stand for chromosomal translocation, deletion, duplication, insertion, inversion, and tumor break load, respectively.

Sample	CTX	DEL	DUP	INS	INV	Total	TBL
Tumor 1 (A23-239)	364	50	30	0	46	490	0.22
Tumor 2 (A24-050)	382	66	16	4	46	514	0.23
Tumor 3 (A23-628)	250	18	16	0	20	304	0.14

### Sample purity, clonality, and mutational burden

**Fig. 2:**
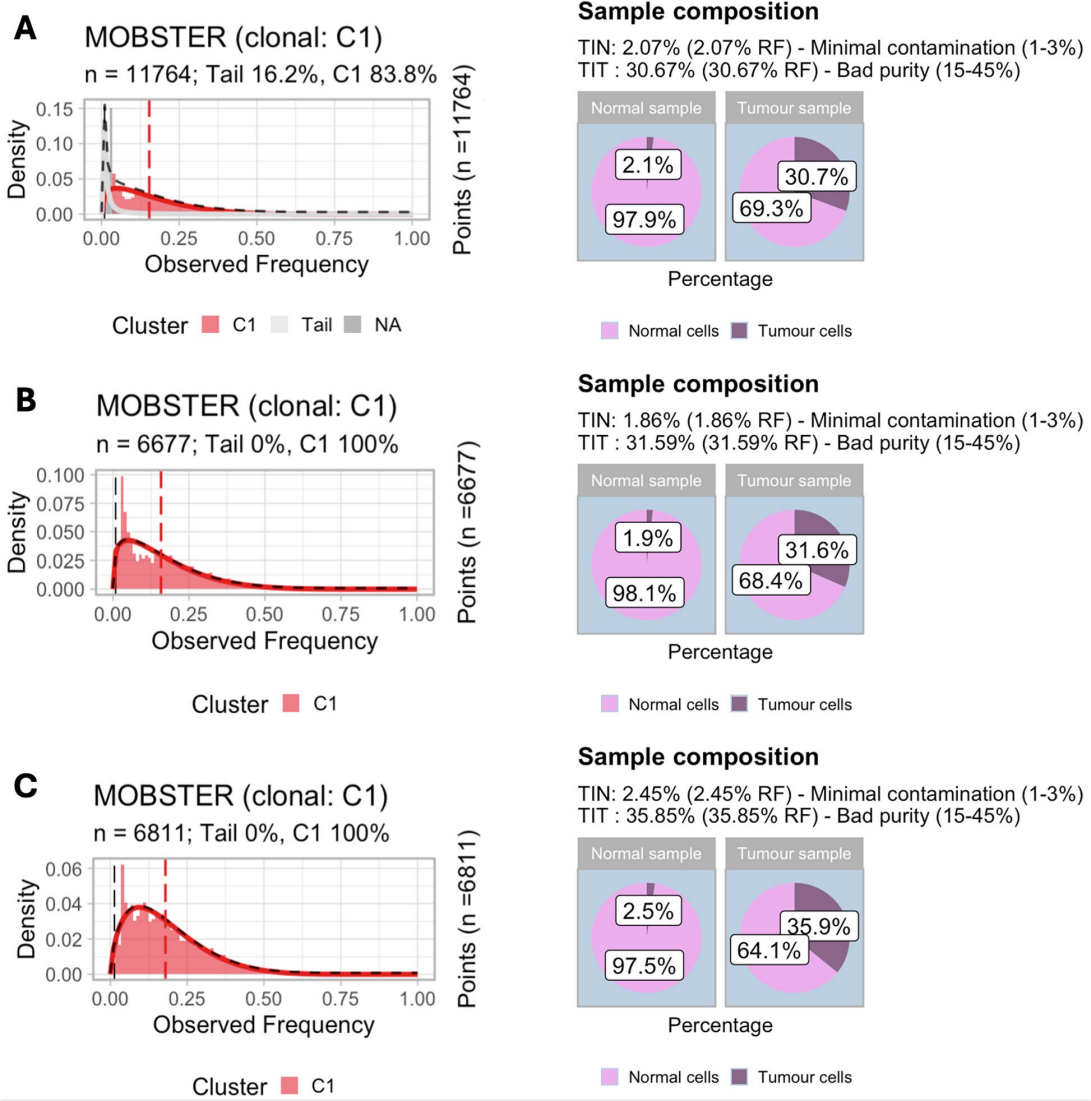
Clonality and purity evaluation for each paired sample. MOBSTER clonal peak detection to estimate multiple clonal populations within a tumor sample (left) and estimated tumor and normal sample purity (right) for each sample, **A** A23-239 (Tumor 1), **B** A24-050 (Tumor 2), and **C** A23-628 (Tumor 3). Dashed red lines indicate the median of a clonal distribution peak. Cluster—an identified clonal cluster, Tail—the identified power law tail (Caravagna et al., 2020 and references within), NA—not assigned to a cluster, TIN—tumor in normal contamination, TIT—tumor in tumor contamination

The three tumor samples analyzed—A23-239, A24-050, and A23-628—showed low tumor purity, ranging from 30.7 to 35.9%, with normal sample contamination levels between 1.86 and 2.45% (Fig. 2). A23-239 (Tumor 1) exhibited the highest total single nucleotide variant (SNV) mutation count at 11,764 (log 4.07), a tumor mutational burden (TMB) of 5.26 mutations/Mb, fraction of genome altered (FGA) of 0.0394, and a tumor break load (TBL) of 0.22 SVs/Mb. A24-050 (Tumor 2) had 6677 total mutations (log 3.82), a TMB of 2.99 mutations/Mb, FGA of 0.0750, and a TBL of 0.23 SVs/Mb. A23-628 (Tumor 3)—the only homozygous for the LF locus in our sample—showed 6811 total mutations (log 3.83), a TMB of 3.05 mutations/Mb, FGA of 0.0219, and a TBL of 0.14 SVs/Mb. Despite differences in mutational burden and FGA, all samples were classified as low purity (14–45%) by TINC and identified as monoclonal by MOBSTER (Fig. 2).

### Genomic mutations

#### Single nucleotide variant (SNV) analysis

To identify potential SNVs contributing to tumorigenesis, we identified SNVs and indels with shared genomic positions present in all tumor samples. We found three point mutations: one in LOC129328665, which encodes zinc finger protein 420-like, one in LOC129328017, which encodes vomeronasal type-2 receptor 26-like, and one point mutation (827C > A) in the TATA-box binding protein (TBP) gene. No insertions or deletions with functional impacts based on *SNPEff* annotations were shared between all individuals.

#### Structural variant (SV) analysis

**Fig. 3:**
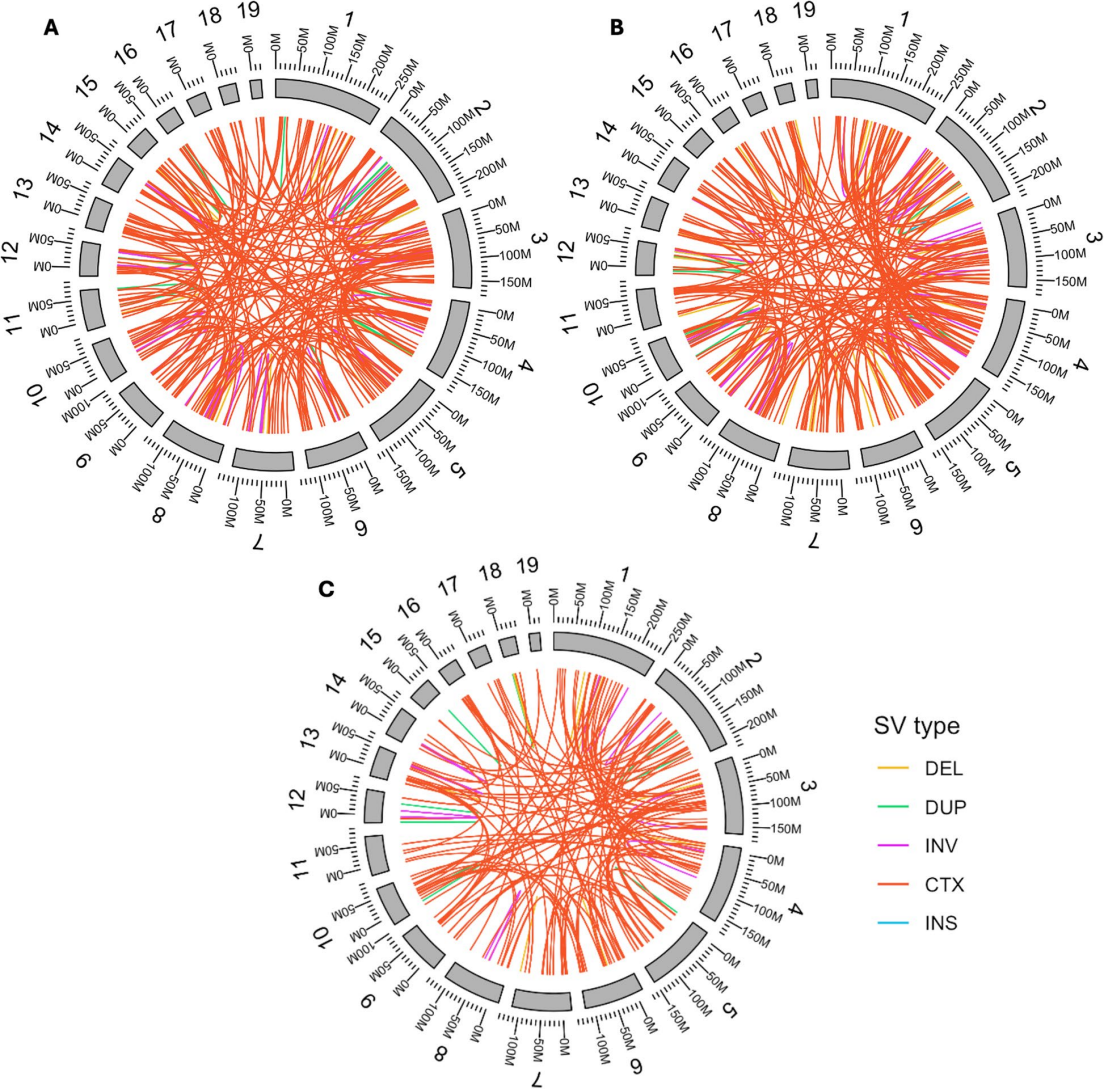
Mapping of structural variant (SV) events by chromosomes. The inner circle is breakpoint mapping within the genome and the outer circle represents the chromosome position. **A** A23-239 (Tumor 1), **B** A24-050 (Tumor 2), and **C** A23-628 (Tumor 3). “CTX,” “DEL,” “DUP,” “INS,” and “INV” stand for chromosomal translocation (red), deletion (green), duplication (blue), insertion (yellow), and inversion (purple), respectively

Chromosomal translocation events were the most common type of SV identified (Table 1) and all tumor samples showed evidence of rearrangements across all chromosomes (Fig. 3). Tumor samples shared 350 genes experiencing a structural variation event, 18 of which are known cancer genes according to the OncoKB Cancer Gene List (Supp. Table 1).

#### Copy number variant (CNV) analysis

Copy number variant events were filtered to a *p* value < 0.05 and a *Control-FREEC* model uncertainty < 20 to take into account tumor purity. We found 33 overlapping genomic regions shared between all three tumor samples—meaning the same gene sets were impacted—that were affected by some form of copy number variation (gain = 7, neutral = 25). From these overlapping regions, a total of 769 currently annotated genes were affected, 46 of which are implicated in human cancers according to the OncoKB Cancer Gene List (Supp. Table 2).

For the copy number neutral loss of heterozygosity (LOH) events, we inspected the SNVs of each sample to identify LOH impacted genes with either HIGH (involves large chromosome segment) or MODERATE (involves one or many codons) mutations according to *SNPEff* annotations; note that this differs from the previous SNV analysis in that the mutations did not necessarily need to be in the same genomic position, only within the same gene. We found moderate impact missense mutations in MAP3K13 (mitogen-activated protein kinase 13), TENM4 (teneurin transmembrane protein 4), and LOC129326353 (olfactory receptor 2AT4-like), meaning that the mutated allele was in homozygosity after the copy number event and potentially resulted in an adverse mutation outcome due to the lack of expression of a wild-type allele. Additionally, we detected four gain events across the genome with high confidence (Supp. Table 2). Gain events involve an allele gain, either as going from homozygotes to heterozygotes or as an increase in the number of genes (and alleles) from the non-tumor samples. Gains were one in chromosome 4 with an amplification of multiple genes within the affected region all encoding for zinc finger like proteins, and one in chromosome 6 with an amplification of a gene encoding for ephrin type-B receptor 5-like protein. Two other regions experienced gain events with currently uncharacterized genes, a 590,625 bp portion of chromosome 1 and a 322,310 bp portion of chromosome 9 (Supp. Table 2). All uncharacterized genes returned no matches to annotated genes based on *BLAST* queries.

### Pathway-level convergent analysis

To identify shared pathway disruptions in the tumor samples, we analyzed all SNV mutations shared across the three tumor samples that were detected to be part of known pathways from GO, KEGG, and Reactome. We identified 12 pathways from the GO dataset and one pathway from the Reactome dataset (Table 2). Of these, eight were implicated in neuronal and brain development, two were implicated in cell growth and size regulation, one in cardiac muscle tissue development, one in muscle cell cytoskeleton regulation, and one in actin filament organization.

**Table 2:** Summary of shared gene pathways altered in tumor samples based on SNVs. *Database* refers to which pathway annotation database returned the result—GO, KEGG, or Reactome. *ID* corresponds to the reference ID within the respective database. All results shown have a Benjamini–Hochberg adjusted *p* value < 0.05

Database	ID	Pathway description
GO	GO:0030900	Forebrain development
GO	GO:0007409	Axonogenesis
GO	GO:0021537	Telencephalon development
GO	GO:0021543	Pallium development
GO	GO:0048738	Cardiac muscle tissue development
GO	GO:0021953	Central nervous system neuron differentiation
GO	GO:0007015	Actin filament organization
GO	GO:0001764	Neuron migration
GO	GO:0007528	Neuromuscular junction development
GO	GO:0042063	Gliogenesis
GO	GO:0048638	Regulation of developmental growth
GO	GO:0032535	Regulation of cellular component size
Reactome	hsa04820	Cytoskeleton in muscle cells

For the pathway-level analysis of CNVs, 190 significant pathways were identified from GO, six from KEGG, and 19 from Reactome (Supp. Table 3). Broadly, this analysis identified multiple pathways that are implicated in cancer such as chromatin organization/remodeling, cell cycle regulation, gene expression/transcription, immune modulation, macromolecule & nucleic acid metabolism, and insulin signaling & metabolic pathways.

## Discussion

This study presents a comprehensive genomic characterization of iridophoroma in the Lemon Frost morph (LF) of the leopard gecko. By comparing primary tumor and non-tumor samples from three individuals, we identify genomic features associated with tumor development. The unique tumor phenotype of this morph highlights its potential as a novel model system for studying the genetic basis of tumorigenesis and metastatic progression. This study was performed using existing bioinformatics tools commonly used for analysis of human cancers, demonstrating the adaptability of existing pipelines to support emerging model organisms.

Genome-wide heterozygosity estimates based on pool-seq data from a previous study [23] identified only slight differences between wild-type, homozygous, and heterozygous individuals at the LF locus. Specifically, genome-wide heterozygosity in homozygous and heterozygous LF individuals was only 0.03–0.04% lower than the wild-type. Our three individuals showed lower genome-wide heterozygosity compared to the pool-seq population data reported by Guo et al. [23], which were based on 63 homozygous and 25 heterozygous LF individuals, but similar values to Pinto et al. [22] from a single heterozygous LF individual (0.35% and 0.38%). Discrepancies in the level of genome-wide heterozygosity may be related to the different numbers of individuals compared in these three studies. The highest measure of genome-wide heterozygosity (0.42% compared to 0.40% and 0.34%) in our samples was found in the homozygous individual for the LF allele; this was also the only individual in our sampling lacking visceral metastases. Although based on a small sample size of only three individuals, this finding may indicate a contributing effect of lower genome-wide heterozygosity on an increased risk of developing visceral metastasis potentially together with homozygosity at the LF allele as previously suggested by Guo and Kruglyak [28]. Note that in our study we did not control for the age of the sampled individuals, although all of them were sexually mature and therefore older than 9–10 months. As pointed out by Guo et al. [23], metastasis can take between 6-12 months of age to develop. Therefore, because the exact age of the homozygous LF male without visceral metastases was not known, it is possible that metastases could have developed later. The lower levels of genetic diversity seen in the LF morph compared to the wild-type population are likely due to inbreeding practices used to establish the LF phenotype [23]. Additionally, the established line of *E. macularius* available in the pet trade, and subsequently in research, already possesses very low genetic diversity, having started from just a few individuals [37]. As the loss of heterozygosity is a typical feature observed in cancer cells and tumorigenesis [38–40], and inbreeding has been observed to be related to lower lifespan and lower fitness [41, 42], decreased genome-wide heterozygosity in the LF morphs could favor the high prevalence of tumors in this organism.

Tumor mutational burden (TMB) rates—a metric used to understand the mutational landscape of tumors and used as a clinical biomarker [43, 44]—in our samples conform to the median TMB rate across multiple human cancer types [45]. TMB in our samples was below 10 mutations/Mb, falling between 2.99 and 5.26; values of TMB > 10 mutations/Mb are considered high [45, 46]. The low TMB identified in our study (TMB < 10 mutations/Mb) reduces the likelihood that tumorigenesis and metastasis are driven by microsatellite instability, DNA polymerase mutations, or defective mismatch repair pathways [45]. Studies characterizing the impact of TMB across a range of human cancers shows a trend that low TMB tumors are less responsive to immune checkpoint inhibitor treatments, have lower patient survival rates [47], and are also typically correlated with reduced neoantigen load [48–50]. Additionally, our findings relating to TMB are similar to those observed in multiple types of childhood cancer [45, 46, 51]. These studies together suggest a greater ability for immune evasion in this tumor type and show potential for the LF as a model for cancer types characterized by low TMB.

A major result of our study is that all studied tumor samples possess a single nucleotide variant in the gene encoding the TATA-box binding protein (TBP) in comparison to non-tumor samples of the same individual, resulting in a threonine-to-lysine substitution at position 276 within its DNA-binding domain. TBP is a conserved subunit of the transcription factor IID complex and is responsible for positioning RNA polymerase II at the transcription start site by binding to the TATA-box motif of TATA-box containing genes. Alignment with the human TBP C-terminal domain, which is highly conserved [52], identified the mutation’s location at position 313-threonine in the human ortholog. Prior studies in humans have shown that mutations in flanking positions (307 and 316) can decrease in vivo function of TBP [53] and therefore impact TBP-DNA binding affinity. This can potentially reduce the expression of TATA-box-containing genes, which represent 10–24% of genes within the human genome [54]. Mutations in the DNA-binding sequence of the TATA-box motif have been shown to disrupt TBP binding and to be associated with hereditary diseases and lung cancer [55, 56]. We also identified shared point mutations impacting the amino acid structure in two other genes, LOC129328665 and LOC129328017. LOC129328665 encodes zinc finger protein (ZFP) 420-like, which is part of a family of proteins that are broadly involved in transcriptional regulation [57] and can regulate cancer cell growth and migration via p53-mediated apoptosis [58, 59]. Mutations in the repetitive zinc finger domains of these proteins have been implicated in several human cancers [60]. Approximately 5% of genes in the human genome encode zinc finger proteins, leading to a large diversification of ZFPs that have different targets for transcriptional regulation [61]. Therefore, the variant within this ZFP encoding gene could have genome-wide impacts during transcriptional processes that can influence transcriptional regulation, chromatin remodeling, DNA repair, and RNA homeostasis in the leopard gecko. Furthermore, LOC129328017 encodes the vomeronasal type-2 receptor (V2R) 26-like, which is a G-protein coupled receptor (GPCR) belonging to an evolutionarily diverse family of genes responsible for chemosensory reception [62, 63]. While there is no evidence that V2R genes exist in humans besides as pseudogenes [64], related olfactory receptors (ORs) are known to be ectopically expressed in human prostate cancers and show a tumor suppressive function [65]. Additionally, they can also function as suppressive regulators of tumor associated macrophages or drive protumor phenotypes in these cells in the presence of palmitic acid [66], which is known to promote metastasis from melanomas [67, 68]. While only one study has directly linked missense variants within human ORs to cancer [69], there is the potential for V2R-mediated disruption of the tumor microenvironment in our system.

Structural variant analysis revealed a high number of events, with considerable variation in the type and size of SVs. Among these events, one putative novel gene fusion—IARS1-RNF213—was shared across all tumor samples. Because this inference is based solely on genomic structural variant data, it does not allow a determination of whether this rearrangement produces a functional fusion transcript or protein product, which would require complementary transcriptomic or proteomic data that were not available for our samples. RNF213 is known to participate in gene fusions in several human cancers, including lung adenocarcinoma, acute myeloid leukemia, and glioblastoma (RNF213-ALK), as well as chronic myeloid leukemia (RNF213-SLC26A11) [70, 71]. RNF213 has been proposed to act as a tumor suppressor in multiple tumor types [70] and fusions observed in transcriptomics analysis of lung cancer has identified it as a likely driver event [72], while knockdown studies in intrahepatic cholangiocarcinoma show that loss of function mutations result in increased cellular migration and invasion activities [73]. Furthermore, IARS1 belongs to the family of aminoacyl-tRNA synthetases (ARSs) that are responsible for multiple cellular processes from their canonical role in protein synthesis to transcriptional regulation, rRNA synthesis, translational silencing, and anti-apoptotic signaling [74]. ARSs have been identified in multiple cancer types, likely selected for due to their diverse roles in cellular function [75]. The fusion of these genes could have novel impacts on diverse cellular functions via a loss of normal tumor suppressive functions or a systemic dysregulation of the cellular metabolome and proteome—in the case of RNF213 and IARS1 functionality, respectively—that could disrupt cellular pathways and promote tumorigenesis. Alternatively, the structural rearrangement may influence tumorigenesis through multiple mechanisms, including the generation of fusion genes, enhancer hijacking leading to aberrant expression of nearby genes, or disruption of regulatory elements that control gene dosage and chromatin architecture. Such mechanisms have been widely documented in cancer genomes, where structural rearrangements can rewire enhancer–promoter interactions or alter 3D genome organization to drive oncogene activation [76–78]. Future studies integrating transcriptomic data and phenotypic assays will be necessary to determine the functional consequences of this recurrent rearrangement and its potential role in tumor development.

Copy number variation (CNV) analysis revealed copy-number-neutral loss of heterozygosity (LOH) in three genes already carrying single nucleotide variants—MAP3K13, TENM4, and LOC129326353 (OR2AT4-like)—highlighting a “double hit” mechanism that can unmask recessive driver events [79]. OR2AT4-like (2AT4) is aberrantly expressed in chronic myelogenous leukemia and has been explored as a therapeutic target to regulate cell proliferation and apoptosis [80]. Although MAP3K13 is not classically oncogenic, it stabilizes key proteins such as c-Myc and mutant p53, positioning it within established cancer-signaling networks when disrupted [81, 82]. TENM4 mutations are recurrent across diverse tumors, where they promote tumor initiation, maintenance, and metastatic cell migration [83, 84]. Together, the coexistence of SNVs and LOH in these loci underscores their potential roles as cancer drivers and suggests that loss of the wild-type allele may amplify the impact of the detected mutations. Additionally, we found a gain in a region on chromosome 4 consisting solely of ZFP encoding genes, which suggests an overexpression of genes regulating transcriptional regulation and chromatin remodeling events. The detection of copy number gain events is likely to drive overexpression of the impacted genes [85, 86]. In humans, amplification of ZFP217 in breast cancer is suggested to act as an oncogene via overexpression of alternatively spliced Kruppel-like transcription factors [87], while ZNF703 overexpression results in epigenetic modifications that promote ovarian cancer proliferation [88]. Ephrin type-B receptor 5-like (EPHB5)—which was found to be amplified on a region of chromosome 6—is part of the Eph receptor tyrosine-kinase family involved in cell-to-cell signaling dynamics that influence cell morphology, adhesion, movement, proliferation, survival, and differentiation [89]. While specific copy number gain events involving these genes are uncommon in the cancer literature, overexpression of multiple Eph gene family members is observed as an oncogene in lung cancer tumorigenesis, where it broadly promotes cell growth and proliferation, colony formation, motility, and migration [90]. Overall, copy-number gains affecting ZFP family members and EPHB5 suggest that increased gene dosage may drive oncogenic overexpression of transcriptional regulators and cell-signaling receptors. Together, these alterations suggest a broad mechanism in which amplified regulators of chromatin, transcription, and Eph-mediated signaling promote tumor growth, plasticity, and metastatic potential.

Finally, we identified a total of eleven dysregulated pathways in tumor samples based on SNVs, with particular emphasis on the disruption of actin filament organization. The disruption of actin filament organization derived from shared SNV-associated pathways suggests genomic dysregulation consistent with mechanisms of metastatic progression [91, 92]. This disruption is consistent with literature on human cancers, especially melanoma, where actin-binding proteins (ABPs) such as WASP, nesprin, and villin contribute to early oncogenic processes by regulating gene expression. SATB1 has been implicated in suppressing apoptosis, while other ABPs and microfilaments support tumor vascularization. Additionally, actin-rich protrusions facilitate cellular mobility, invasion, and metastasis, highlighting the central role of cytoskeletal remodeling in cancer development [93].

Because samples were obtained opportunistically from available clinical cases, our sampling strategy was not designed to systematically compare tumors across developmental stages or anatomical locations. Consequently, the present study cannot directly resolve patterns of tumor evolution within individuals. Future work comparing tumors of different sizes and anatomical sites within the same animals, and across individuals sharing the same LF genotype, would provide important insight into tumor progression and clonal dynamics. The genomic alterations identified here therefore represent candidate loci that can guide targeted functional and evolutionary analyses in future studies.

## Conclusions

In this work, we found several mutations likely to affect genome-wide transcriptional regulation, including variants in TBP and ZFP420, copy number gains of multiple ZFP genes, and the novel IARS1–RNF213 fusion. Both TBP and IARS1 are involved in cellular stress responses [75, 94], which are known to promote tumor survival within the tumor microenvironment (TME) [95]. Furthermore, the detected copy-number LOH alterations may contribute to the early establishment and persistence of tumors, particularly through effects on DNA repair and apoptosis pathways [96]. These findings point toward several avenues for future research, spanning well-established cancer mechanisms across species—as seen for copy-number alterations—as well as potentially novel drivers, such as the TBP mutation and IARS1–RNF213 fusion. To note, tumor purity in our samples was low and this could impact the accuracy of copy number calling algorithms [97, 98]. As such, candidate regions of copy number alterations presented here should be followed up with single cell sequencing to improve sample purity [99].

Although metastasis is a documented feature of the LF morph [23, 28], the direct assessment of metastatic progression will require longitudinal and multisite sampling to track tumor evolution through time. As genomic data from this tumor type and related reptilian neoplasms are limited, broader genomic profiling will be critical to determine baseline tumor characteristics and to investigate the TME in greater depth. In addition, assessing transcriptomic consequences of the genomic alterations reported here—particularly through RNA expression, splicing analyses, and protein profiles—may reveal further cancer-relevant mechanisms, as demonstrated in other systems (e.g., splice variants of heparanase in the blind mole rat; [16]). However, RNA extraction challenges have previously hindered studies in this model [23], and improvements to these techniques will be necessary. Finally, targeted sequencing of key candidate loci such as TBP in larger cohorts may clarify their role in tumor initiation and progression.

Overall, this study establishes a genomic foundation for understanding iridophoroma in the LF leopard gecko and demonstrates the translational potential of this morph as a research model. Studying tumor development in a naturally occurring, untreated system allows the exploration of cancer biology without therapeutic selection pressures [100], capturing genomic events that may be conserved across vertebrates. The LF morph thereby offers a unique, accessible, and biologically relevant model for investigating both well-characterized and emerging mechanisms of tumorigenesis and metastasis.

## Methods

### Animal care and samples

The three leopard geckos from which samples were collected were all LF morphs, either heterozygous or homozygous for the LF allele. Two of the geckos were housed at George Mason University (VA, USA) in the Chiari Lab and one at Marquette University (WI, USA) in the Gamble Lab. Housing conditions can be found in White et al. (2024). Individuals were monitored weekly for typical visible skin discoloration and growths (Fig. 1) and for signs of decreased quality of life (i.e., lethargy, poor appetite, decreased mobility). If any of these signs appeared, individuals were euthanized at their respective animal care facility using tricaine methanesulfonate following the procedures of Conroy et al. [101]. Euthanized individuals were then packed with ice packs in a box and shipped within 24 h to the University of Florida College of Veterinary Medicine for necropsy and microscopic examination.

Individual A23-239 (Tumor 1) was an adult male heterozygous for the LF allele mutation. Neoplastic nodules were associated with the integument of the head, body, and limbs externally. Visceral masses were observed in the liver. The neoplasms consisted of mixed morphology chromatophores, with microscopic features of both iridophores and erythrophores. Individual A24-050 (Tumor 2) was a 19-month-old female heterozygous for the LF allele. Neoplastic nodules were associated with the integument of the head and body. Visceral masses were observed in the kidneys. The neoplasms consisted of homogenous chromatophores with features of iridophores. Individual A23-628 (Tumor 3) was a male of unknown age homozygous for the LF allele (super lemon frost). Neoplastic nodules were associated with the integument of the head, body, and tail. Visceral masses were not observed, but masses were present in the eyes (irides). Heterozygosity and homozygosity at the LF locus were based on the genomic data collected in this study and mutations associated with the LF alleles on Scaffold996 of the draft leopard gecko genome [23, 102].

The neoplasms consisted of homogenous chromatophores with features of iridophores. Non-tumoral tissues were sampled at least 1 cm away from a focus of gross iridophore proliferation (tumoral tissue); half of the tissue was frozen and the other half was assessed microscopically for absence of neoplasia. Samples of neoplastic tissue (primary tumor) and non-neoplastic tissue distal to tumors were collected for each individual and stored at − 80 °C until shipping.

### Sample preparation and sequencing

Primary tumor and non-tumor tissue samples were extracted from each of the three individuals. Samples were shipped in dry ice to the Genomics and Cell Characterization Core Facility (GC3F) at the University of Oregon (OR, USA), where DNA extractions were carried out following the Thermo Scientific KingFisher method. Tissue was extracted using a Mag-Bind Blood & Tissue DNA HDQ Prefilled 96 Kit following manufacturer’s protocol [103]. Quality of extracted DNA was measured using Nanodrop, Qubit, and fragment analysis using the Agilent HS Large Fragment Kit. DNA was sheared using Covaris (Covaris, Woburn, MA, USA) to a target insert size of 550 bp. Sheared DNA was ligated to stubby partial adapter using Watchmaker DNA Prep kit (Watchmaker Genomics, Boulder, CO, USA) and amplified for 3 cycles with barcoded primers containing unique dual 10 bp indexes. Finished libraries were tested with fragment analysis and qPCR to ensure appropriate library size and concentration prior to pooling and sequencing. Whole-genome sequencing was conducted on the tumor and non-tumor samples using Illumina NovaSeq 6000: S4 Single Lane of Paired-End 150 nt at 100 × coverage.

### Alignment of reads and somatic variant analysis

A genomic analysis to detect somatic variants within the tumor samples was done using the *Sarek* bioinformatics pipeline version 3.4.4 [104, 105] available from nf-core, a shared repository of bioinformatics workflows [106]. Specifically, all samples were aligned to the *E. macularius* reference genome that was previously assembled from a heterozygous LF individual [22] (NCBI RefSeq assembly: GCF_028583425.1) using *BWA-mem* v0.7.18 [107] from paired-end FASTQ files (batch size = 100,000,000, mismatch penalty = 3, with soft clipping for supplementary alignments). Duplicates were marked in *GATK Markduplicates* v 4.5.0.0. The pipeline also included quality checks for sequencing files using *FastQC* v 0.12.1 and *FastP* v 0.23.4 with quality checks for the aligned sequence files using *Samtools* v 1.2, *Mosdepth* v 0.3.8, and *GATK Picard* v 4.5.0.0. These reports were summarized into a collective report of all samples from all reporting tools using *MultiQC*.

Paired sample variant calling between the tumor and non-tumor sample from each individual was done to identify single nucleotide variants (SNVs) along with insertions and deletions (indels), structural variants (SVs), and allelic copy number variants (CNVs) using *Strelka2* [108], *Manta* [109], and *Control-FREEC* [110], respectively. *BLAST* queries (*BLASTn* v 2.17.0) with default parameters were conducted for any non-annotated genes returned during analyses [111].

**Fig. 4:**
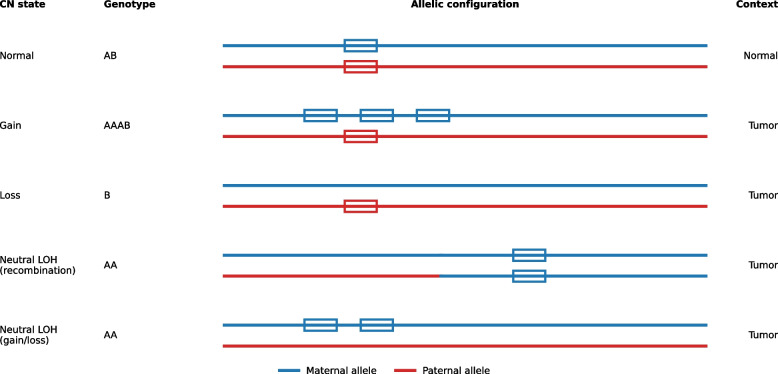
Illustration of allelic configurations associated with copy number variation (CNV) states in cancer. Schematic representation of major CNV states and their resulting genotypes that could be encountered in tumor versus normal cells (*Context*). Maternal and paternal homologous chromosomes are shown in blue and red, respectively; boxed regions denote the genomic locus of interest. The *normal* diploid state contains one copy of each parental allele (AB). *Copy number gain* results from focal (one specific gene is interested) or regional (a chromosomal fragment) amplification, leading to additional copies of one allele (e.g., AAAB). *Copy number loss* corresponds to deletion of one parental allele (B). *Copy-neutral loss of heterozygosity (LOH)* can arise through mitotic recombination, producing two identical alleles (AA) without a net change in copy number, or through combined gain and loss events. Although copy number remains diploid in neutral LOH, allelic diversity is lost

Copy number variant (CNV) identification tools often require a list of known single nucleotide polymorphisms (SNPs) to estimate allelic frequency at known heterozygous sites. This information is used to identify challenging regions with allelic imbalance, such as copy number neutral loss of heterozygosity (LOH) regions [110], where one allele is lost and the remaining allele is duplicated, resulting in a change from a heterozygous to a homozygous state. This genomic variation is considered neutral because the diploid status in that region is not changed. CNV includes any variation in allele number between tumor and non-tumor samples. As such, CNV is reflected in changes from heterozygosity to homozygosity (and vice versa), but also by gene loss or duplication (Fig. 4). For this analysis, non-tumor samples from the current study and pool-seq samples of saliva from a previous study [23] (NCBI accession number PRJNA730084) combining DNA from 159 individuals were used to identify shared SNPs needed to increase copy number identification accuracy. The 159 individuals from the previous study included 71 wild type, 63 lemon frost, and 25 super lemon frost (homozygous individuals at the LF allele) [23], with pool-seq samples collected for each group separately (respective SRA run IDs: SRR14552569, SRR14552567, SRR14552568). These samples were downloaded from the NCBI SRA as paired-end FASTQ files and subsequently aligned to the leopard gecko genome as described above. Variant calling for germline SNVs from these samples was carried out using *Freebayes* [112] for each pool-seq sample as it has been successfully used previously to call variants in pooled samples [113]. The variant call format (VCF) files of pool-seq, along with the germline variants detected in the present study using *DeepVariant* [114], were concatenated using *bcftools concat* with duplicate positions removed to create a single VCF file of known germline SNPs, then filtered to subset the reads with QUAL > 20 and allele depth (DP) > 10, for a total of > 20 million germline variant sites relative to the reference genome, which was used as input for known SNP positions during copy number calling (for the CNV analysis).

Germline variants from the current study and Guo et al. [23]—called using *DeepVariant* and *Freebayes*, respectively, as described above—were also used to estimate the heterozygosity of the genome separately for the data of our study and Guo et al. [23], which was analyzed separately for wild-type, heterozygous, and homozygous populations as defined in their study. Briefly, germline variants were filtered with QUAL > 20 and a variant allelic frequency (VAF) < 90% to retain only heterozygous germline SNPs, which were then divided by the total genome length to obtain the genomic heterozygosity measure.

Additionally, tumor samples are often impure and thus appear as a mixed tumor-normal signal when inferring copy number states. To correct for this, *Control-FREEC* supports an estimate of tumor purity as input to adjust its segmentation and copy number modeling and infer true tumor-specific CNVs [110]. We used the software package *TINC* [115] to estimate tumor purity levels based on the SNVs identified in each tumor sample, which gives a measure of the tumor purity as tumor in tumor (TIT) and a measure of the normal sample purity as tumor in normal (TIN). As the copy number calling only allows one input parameter for tumor purity when running samples in parallel, the a priori tumor purity estimate was an average of the three tumor samples (mean = 32.7%, SD = ± 2.8%). Finally, tumor clonality was evaluated with *MOBSTER* [116] to detect clonal peaks based on the SNV analysis of each sample.

### Single nucleotide variation (SNV) and pathway-level analyses

Using *Strelka2* somatic SNV calls annotated with *SNPEff* [117], we subset the calls to PASS mutations with a MODERATE or HIGH functional protein impact, as defined by *Strelka2* and *SNPEff*, respectively. For pathway-level analysis, the resulting transcript IDs of the gecko were mapped to their protein IDs, which were then mapped to human orthologs using *DIAMOND* [118]. Pathway enrichment was done for GO [119, 120], KEGG [121], and Reactome [122] pathways for each sample individually. The resulting enrichments were then analyzed for significant overlap to observe any potential convergence of pathway dysregulation in the tumor.

A modified method was used to analyze pathway-level impacts from CNVs (copy number variations), where shared CNV regions were identified across samples before pathway-level analysis. The resulting genes were then run within GO, KEGG, and Reactome to determine biological pathways that may be impacted. Specifically, CNV-impacted genes identified as cancer relevant in the OncoKB gene list were used as the genes of interest, while all of the genes that were identified in the overlapping regions—including the previously mentioned cancer-relevant genes—were used as background genes in the pathway enrichment tests to isolate biological signal specific to cancer-relevant CNV targets, rather than the whole genome.

### Metrics for genomic impact of tumors

For each of the variant calling steps mentioned (SNV, SV, and CNV), metrics were calculated to evaluate the impact of the somatic tumor mutations based on previous literature. Tumor mutational burden (TMB) is the number of somatic mutations per megabase of DNA in the tumor genome [123], taken from SNV data. Tumor break load (TBL) is a measure of structural variants in a sample relative to genome size, useful as a measure of genome instability within samples and across tumor types [124], taken from SV data. Fraction of genome altered (FGA) is the proportion of the genome affected by copy number variations [125], taken from CNV data.

## Supplementary Information

Supplementary Material 1

## Data Availability

Raw sequencing reads generated and analyzed during the current study are available from NCBI Sequence Read Archive (Accession number PRJNA1339012). Scripts used in the analyses are available on GitHub (https://github.com/brandon-hastings/LF_tumor_analysis ).
